# Poly(adenosine diphosphate-ribose) polymerase as therapeutic target: lessons learned from its inhibitors

**DOI:** 10.18632/oncotarget.16859

**Published:** 2017-04-05

**Authors:** Anna Mária Cseh, Zsolt Fábián, Balázs Sümegi, Luca Scorrano

**Affiliations:** ^1^ Department of Biochemistry and Medical Chemistry, University of Pécs Medical School, Pécs, Hungary; ^2^ Department of Biology, University of Padova, Padova, Italy; ^3^ Conway Institute, University College Dublin, Belfield, Dublin, Ireland

**Keywords:** PARP, mitochondria, cancer, signaling, targeted therapy

## Abstract

Poly(ADP-ribose) polymerases are a family of DNA-dependent nuclear enzymes catalyzing the transfer of ADP-ribose moieties from cellular nicotinamide-adenine-dinucleotide to a variety of target proteins. Although they have been considered as resident nuclear elements of the DNA repair machinery, recent works revealed a more intricate physiologic role of poly(ADP-ribose) polymerases with numerous extranuclear activities. Indeed, poly(ADP-ribose) polymerases participate in fundamental cellular processes like chromatin remodelling, transcription or regulation of the cell-cycle. These new insight into the physiologic roles of poly(ADP-ribose) polymerases widens the range of human pathologies in which pharmacologic inhibition of these enzymes might have a therapeutic potential. Here, we overview our current knowledge on extranuclear functions of poly(ADP-ribose) polymerases with a particular focus on the mitochondrial ones and discuss potential fields of future clinical applications.

## INTRODUCTION

Basal metabolic activity as well as environmental factors lead to more than 20,000 DNA alterations per cell every day [1]. Since structural DNA damage may interfere with transcription and, consequently, disarray proteome homeostasis, maintenance of genomic integrity is critical for cellular function. Genomic maintenance primarily depends on a network of different repair mechanisms, collectively termed DNA damage response (DDR). Decades of intense research revealed that one of the DDR pillars is the poly-adenosine diphosphate-ribose polymerase (PARP). Based on its central role in the nuclear repair machinery, PARP has been considered as a potential target in cancer cells with compromised repair machinery. According to this concept, inhibition of PARP acts as a “second hit” on the malfunctioning cellular repair armament of, for instance, BRCA1/2-negative cancers resulting in fatal chromosome instability, cell cycle arrest and apoptosis. This led to the development of therapeutic PARP inhibitors and the idea of their use to potentiate antineoplastic alkylating agents.

Although PARP inhibitors, like Olaparib or Veliparib, failed to deliver expected results in clinical trials, they led to the discovery of a number of PARP-mediated extranuclear effects including mitochondrial functions or crosstalk with canonical signaling pathways [2–4]. Here, we overview our current understanding on the mechanism of action of PARP and its pharmacological inhibitors with particular attention to their clinical relevance.

### PARP

**Figure 1:**
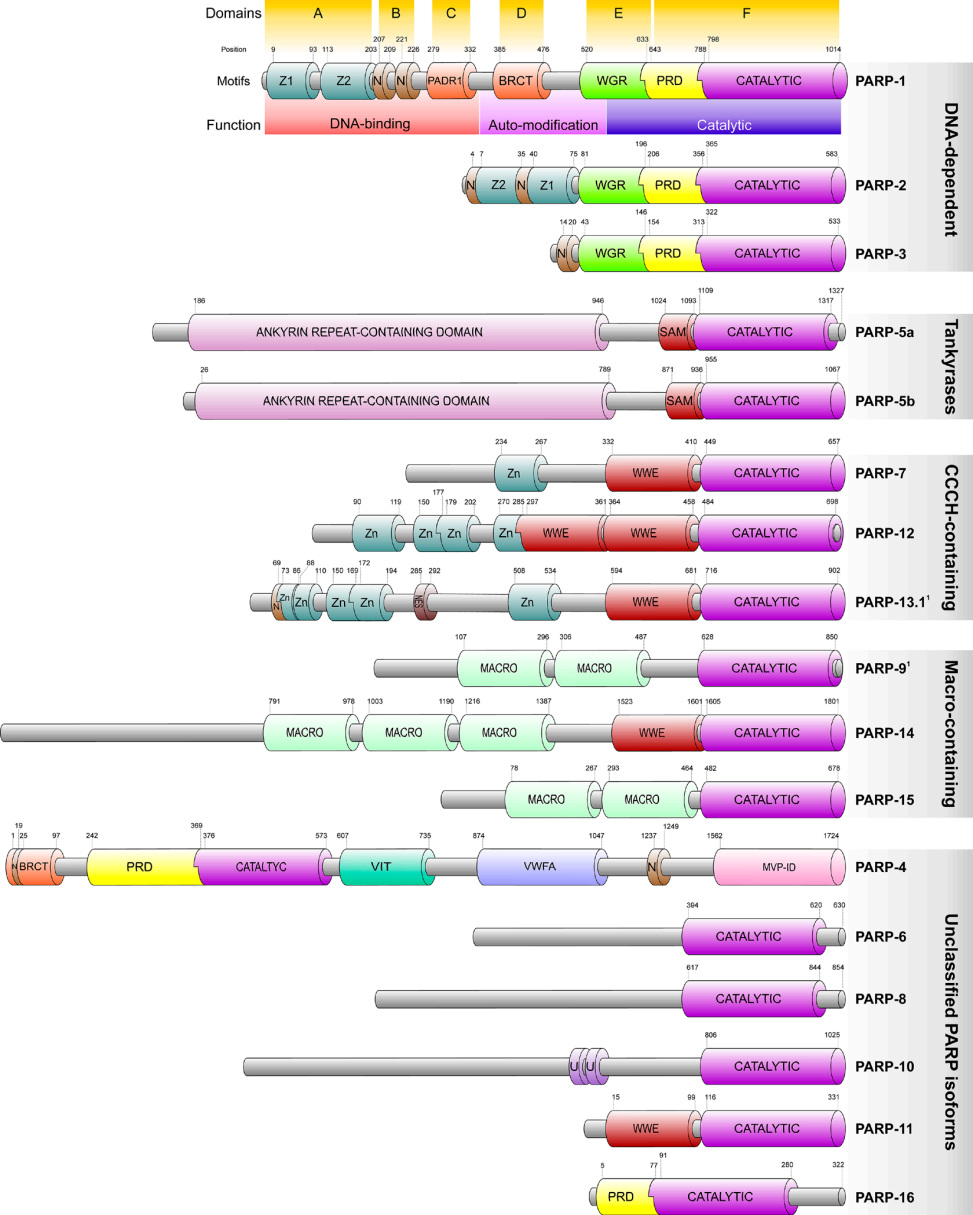
Domain structure and classification of the human PARP polypeptides Figure is based on the Uniprot, RCSB and InterPro databases. Members are related on the basis of the presence of the conserved PARP catalytic domain (CATALYTIC) typically located at the C-terminus of the polypeptides and is characteristic of all PARP protein family members. Within the catalytic domain, the active site is formed by a block of 50 amino acids which is strictly conserved among the vertebrates and highly conserved among all species. Proteins are not drawn to scale but typical structural limits and positions are indicated. ^1^PARP-9 and -13.1 are believed to be catalytically inactive. The alternative isoform of PARP-13 (PARP-13.2) is not indicated in the figure due to its complete lack of the PARP catalytic domain. Labelled structures are follows: Z, Z1 and Z2: PARP-type zinc finger domains; N: Nuclear localization signal; NES: Nuclear export signal; BRCT: breast cancer susceptibility gene associated C-terminal domain; WGR: It is a domain with unspecified functions present in a number of PARPs and named after its most conserved central motif.; PRD: PARP regulatory domain, consists of a duplication of two helix-loop-helix structural repeats and is typically associated with the C-terminal catalytic domain.; SAM: Sterile Alpha Motif, also known as helix-loop-helix domain, exhibits a conserved structure involved in interactions with proteins, DNA and RNA.; WWE: The WWE domain is named after three of its conserved residues and believed to serve as an interaction module.; MACRO: The Macro domain is a 180 amino acids long region that mediate ADP-ribose binding associated with catalytic domains of PARP or sirtuins.; VIT: Vault Protein inter-alpha-trypsin domain; VWFA: von Willebrand factor type A domain that mediates metal ion-dependent adhesion of partner proteins.; MVP-ID: Major Vault Protein interaction domain; U: ubiquitin-binding motif.

Poly(ADP-ribose) polymerases play pivotal role in the intricate network of intracellular processes counteracting genetoxic stress in higher eukaryotic cells [5]. PARP was first identified in 1963 as a nuclear enzyme responsible for the majority of poly(ADP-ribosyl)ation activity [6]. Although PARP-1 is the most abundantly expressed isoform, multiple PARP-encoding sequences have been identified in the human genome [7]. Analyses of transcript abudance in epithelial cell lines of various tissues revealed that, apart from PARP-15, all *PARPs* are expressed [8]. According to the currently available structural data, all 17 known PARP polypeptides, with the exception of PARP-4, have a conserved C-terminal catalytic domain. In contrast, they display great variability of various domains and functional motifs in their N terminus. These include regulatory and binding motifs (e.g. the PARP alpha helical motif or PRD), localization signals, specific regions responsible for interaction with partner molecules (e.g. the WWE domains in PARP-7, the Myc-binding region in PARP-10 or the HIF1AN-binding region in PARP-5b), regions of compositional bias (e.g. the poly-Histidine, Proline- or Serine-rich regions in PARP-5a) or the zinc finger and ubiquitin-binding domains. Although the relevance of the N-terminal domain structure variance is still not clear, it suggests differential physiologic roles and, potentially, spatio-temporal control of the PARP isoforms and represents the basis of their classification into one of the five subgroups of the family (Figure 1).

Poly(ADP-ribosyl)ation is catalyzed by the DNA-dependent and tankyrase PARPs as well as the unclassified PARP-4. The majority of the other isoforms, however, perform mono(ADP-ribosyl)ation with the exceptions of PARP-10 which has poly(ADP) transferase activity, and PARP-9 and -13 that are believed to be catalytically inactive [9, 10]. While intracellular localisation of the PARP family members varies during cell cycle, PARP-1 was shown to be predominantly nuclear. PARP-1, -2 and -3 are ubiquitously expressed in mammalian tissues and are the only DNA strand break-activated isoforms identified so far [11–13].

PARP-1, the best characterised member of the PARP family, is a 116 kDa protein composed of 6 main domains (domain A to F) each with distinct functions (Figure 1) [14]. Domain A functions as part of the DNA-binding module (DBM) and is responsible for the recognition of damaged DNA loci via two zinc-finger motifs. Domain B spans a bipartite nuclear localisation signal that directs PARP-1 into the nucleus and serves as a caspase-3 cleavage site [15]. Similar to domain A, domain C contains a zinc-binding motif (PADR1) that facilitates formation of the DNA-activated conformation of PARP-1 via interdomain contacts. Although not required for physical DNA binding, its absence compromises the catalytic activity of PARP-1 upon DNA binding. Domain C together with Domain A and B are believed to form the N-terminal DNA-binding module of PARP-1 [16]. Domain D mediates negative auto-regulatory post-translational modifications via glutamate, aspartate and lysine residues that reduce enzymatic activity leading to relaxed DNA binding [17, 18]. Another region of Domain D, termed Breast Cancer-Associated 1 C-terminal domain (BRCT), acts as a binding interface for various nuclear partners [19]. Domain E and F together form the catalytic site that binds nicotinamide adenine dinucleotide (NAD^+^) and catalyses poly(ADP-ribosyl)ation also termed as PARylation [20]. In addition, the catalytic domain promotes localized compaction of chromatin into supranucleosomal structures through interaction with the DNA binding domain independently of its enzymatic activity [21].

Although DNA single-strand breaks are the primary stimuli for PARP-1, its activity has also been reported in the absence of DNA lesions. Indeed, PARP-1 recognizes unusual DNA conformations including cruciformed or supercoiled structures as well as bent or stably un-paired DNA regions [22–24]. In support of these observations, PARP-1 is activated in response to a wide range of stimuli that potentially affect DNA conformation including oxidative agents, ethanol, DNA alkylation, excitotoxic injury, lipopolysaccharides (LPS), elevated extracellular glucose concentration or vitamin A depletion [25–31]. Since PARP-2 accounts for approximately 15% of the cellular poly(ADP)-ribose only, PARP-1 is believed to be responsible for the majority of cellular PARylation. Similar distribution of their cellular targets further supports the idea that PARP-1 is the primary PARylating entity [32].

Upon activation, the DBM scans and directs PARP-1 to damaged DNA loci by recognizing aberrant DNA conformations or disruptions of the sugar-phosphate backbone [12]. Activated PARP-1 cleaves NAD^+^ into nicotinamide and ADP-ribose and covalently attaches 50–200 ADP-ribose units to target molecules through glutamate, aspartate or lysine residues [6, 18]. At damaged DNA loci, auto-PARylation triggers recruitment of repair enzimes and, consequently, initiates DNA repair [33]. Being negatively charged, poly(ADP-ribose) alters the biochemical properties of the acceptor molecules resulting in the modulation of their structure, function or localization [34]. Indeed, due to their physico-chemical properties, initially synthesized long and branched PAR chains repell chromatin from the vicinity of the damaged locus preventing accidental homologous recombination between the broken backbone and neighbouring chromatin sections. These initially formed PAR chains, however, are trimmed back to short ones by poly(ADP-ribose) glycohydrolase (PARG), that persist on auto-modified PARP-1 even after its release from DNA. This persistent auto-PARylation prevents PARP-1 to rejoin damaged DNA that might inhibit the execution of the repair process [35].

Elevated NAD^+^ consumption and PAR levels are both observed upon treatments with DNA alkylating agents or ionizing irradiation supporting the idea that PARP-1-mediated PARyaltion is part of the DDR [5]. Indeed, a number of repair molecules harbour PAR-binding motifs including elements of the base-excision repair (BER) [36]. The BER acts on single nucleotide lesions catalysing excision and replacement of damaged or incorrectly incorporated nucleotides [37]. As part of the BER, PARP is believed to bind DNA strand-breaks and by simultaneous auto- and target PARylation facilitates the recognition and repair of affected loci. The nature of the interplay between PARP-1 and elements of the BER, however, is still obscure due to apparently conflicting experimental results. Although PARylation facilitates recruitment of XRCC1, the scaffold molecule that recruits additional BER enzymes to damaged foci, BER remains intact even in PARP-1 negative cells [38–40]. In addition, while they show increased sensitivity toward PARP inhibitors, XRCC1-depleted cells exhibit intact BER activity suggesting a BER-independent role of the PARP-1-XRCC1 interaction [41]. To date, the most widely accepted scenario is that PARP-1, perhaps via XRCC1, contributes to certain types of the BER depending on the nature of the insult or intermediates formed but it does not play an indispensible role in the process reflecting the existing redundancy in the BER system [42].

In the nucleotide excision repair (NER), PARP-1 PARylates DDB2, an essential protein for recognition and removal of UV-induced DNA lesions. PARylation of DDB2 prolongs its chromatin retention time and recruitment of the chromatin remodeler ALC1 that facilitates nucleosome sliding and recruitment of further NER proteins like XPC [43]. Interaction of PARP-1 with various elemets of the mismatch repair has also been reported. These include replication protein A, replication factor C and the proliferating cell nuclear antigen (PCNA) as well as the MutSα-exonuclease 1 (EXO1) complex. The interplay between PARP-1 and the MutSα-activated EXO1 complex results in enhanced 5′-directed excision activities by, possibly, repressing EXO1-mediated hydrolysis, preferentially, on homoduplex DNA [44].

In addition, pharmacological inhibition of PARP-1 was found to result aberrant activation of the non-homologous end joining repair (NHEJ) as well suggesting its role in the repair of double-strand breaks. In support of this concept, not only DNA-dependent protein kinases were identified in the PARP-1 interactome but PARylation was also found to increase their kinase activity [36, 45].

**Figure 2:**
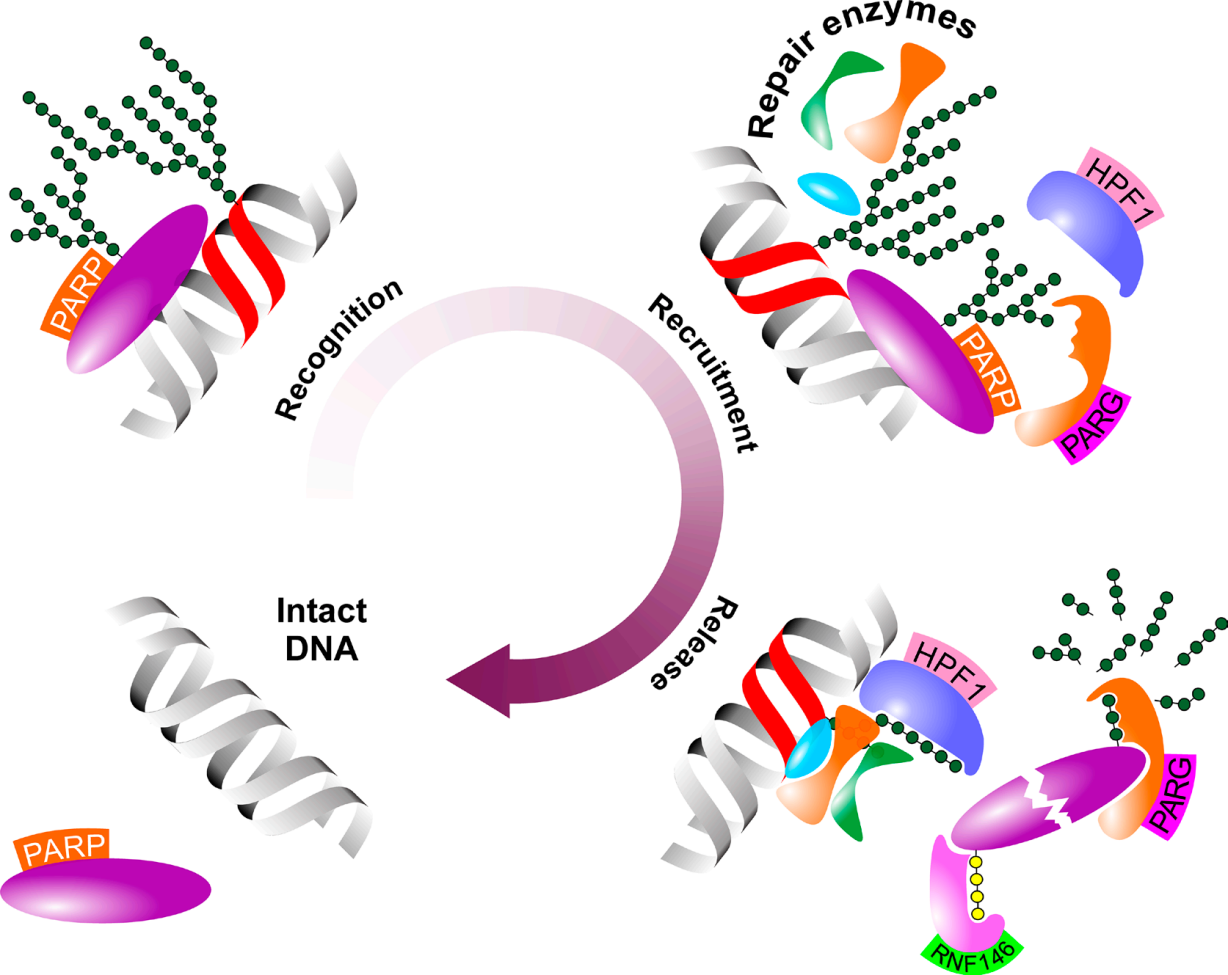
Schematic figure of the role of PARP-1 in the DNA demage response PARP contributes to the DDR in three major steps upon DNA damage. First, PARP scans and identifies dmaged DNA loci (Recognition). Second, PARP-catalysed PARylation attracts repair elements to the damaged DNA locus (Recruitment). Finally, de-PARylation detaches PARP from partner molecules and facilitates its proteasomal degradation (Release). Abbreviated elements are: PARP: Poly(adenosine diphosphate-ribose) polymerase-1; HPF1: Histone PARylation factor 1; PARG: poly(ADP-ribose) glycohydrolase; RNF146: RING finger protein 146. Green and yellow polymers represent PAR and polyubiquitin chains, respectively.

A critical prerequisite for the catalytic activity of recruited repair enyzmes is termination of PARylation and dissociation of PARP-1 from affected loci. In this process first, PARP-1 auto-PARylates within domain D that results in the release of PARP-1 from target molecule, a critical step in the abortion of target PARylation. Apparently, one of the critical regulators of this step is the recently described Histone PARylation factor 1 (HPF1) that controls retention time of PARP-1 via regulation of both ADP-ribosylation of histones and auto-modification of PARP-1 [46]. Once released, PARP-1 is ubiquitinated by the PAR-dependent E3 ubiquitin ligase RING finger protein 146 rendering PARP-1 for proteasomal degradation [47]. Finally, removal of polymerized PAR flags are mediated by dedicated enzymes PARG and poly(ADP-ribose) hydrolase 3 that catalyse degradation of PAR within minutes [48, 49] (Figure 2).

Intensive research on PARP-1's mechanism of action in DNA repair led to the concept that defective PARP-1 functions result in genomic instability and cell death fueling the idea of the use of pharmacological PARP-1 inhibitors in anti-cancer therapies [50].

### PARP inhibition in cancer treatment

The first *in vitro* experiments with distinct PARP inhibitors concluded that these compounds are more potent in BRCA-deficient cellular models and exert synergistic effects when used in combination with chemotherapeutics like the alkylating agent temozolomide or the topoisomerase inhibitor topotecan [51–53]. Considering the central role of BRCA-1 and -2 in DNA repair, these observations underpinned the idea that PARP inhibition primarily targets repair capacity. It is noteworthy however, that other studies reported no or only cell type-dependent effects of PARP inhibitors when tested in combination of platinum agents [54, 55]. More interestingly, some studies reported that excessive PARylation and sustained existence of PARP-DNA complexes are more cytotoxic than genetic depletion of PARP suggesting a more versatile role of PARP-1-mediated PARylation [56].

*In vivo* studies using PARP inhibiting compounds in combination with chemotherapeutics led to similar conclusions. Using the PARP inhibitor compound AG14361 in a human colon tumor xenograft model, improved therapeutic index was reported when used in combination with temozolomide, irinotecan or irradiation [57]. Veliparib, also known as ABT-888, was found to enhance the effect of temozolomide in various xenograft models in a dose-dependent manner leading to increased survival rates [58]. Similarly, Olaparib, also known as compound AZD2281, showed synergistic effects with cisplatin and carboplatin in a BRCA1-deficient mammary tumor model resulting in prolonged survival [59, 60]. In the theet of encouraging preliminary experimental data on pharmacological inhibition of PARP-1, clinical trials delivered rather inconsistent results. While a phase I trial reported significant antitumor activity in Olaparib-treated patients suffering from BRCA mutation-harbouring breast, ovarian and prostate cancers, another study found no synergistic effect between Olaparib and dacarbazine in chemonaive melanoma patients [61, 62]. Similarly, while Olaparib was reported effective in ovarian cancer patients in a phase II trial, the same study declared it inefficient in breast cancer patients independently of their BRCA status [63].

Controversal clinical results raised the question whether the capacity of PARP inhibitors to sensitize cells to chemotherapeutic agents is dependent on the therapeutic context and restricted to certain cell types or chemotherapeutic compounds. Indeed, to date, PARP inhibition has only been approved for monotherapy of BRCA mutation-harbouring neoplasms [64]. Contradicting data on the use of PARP inhibitors, however, raised the possiblity of more intricate underlying functions of PARP that might influence cellular physiology beyond repair.

### PARP-1 as a mitochondrial regulator

**Figure 3:**
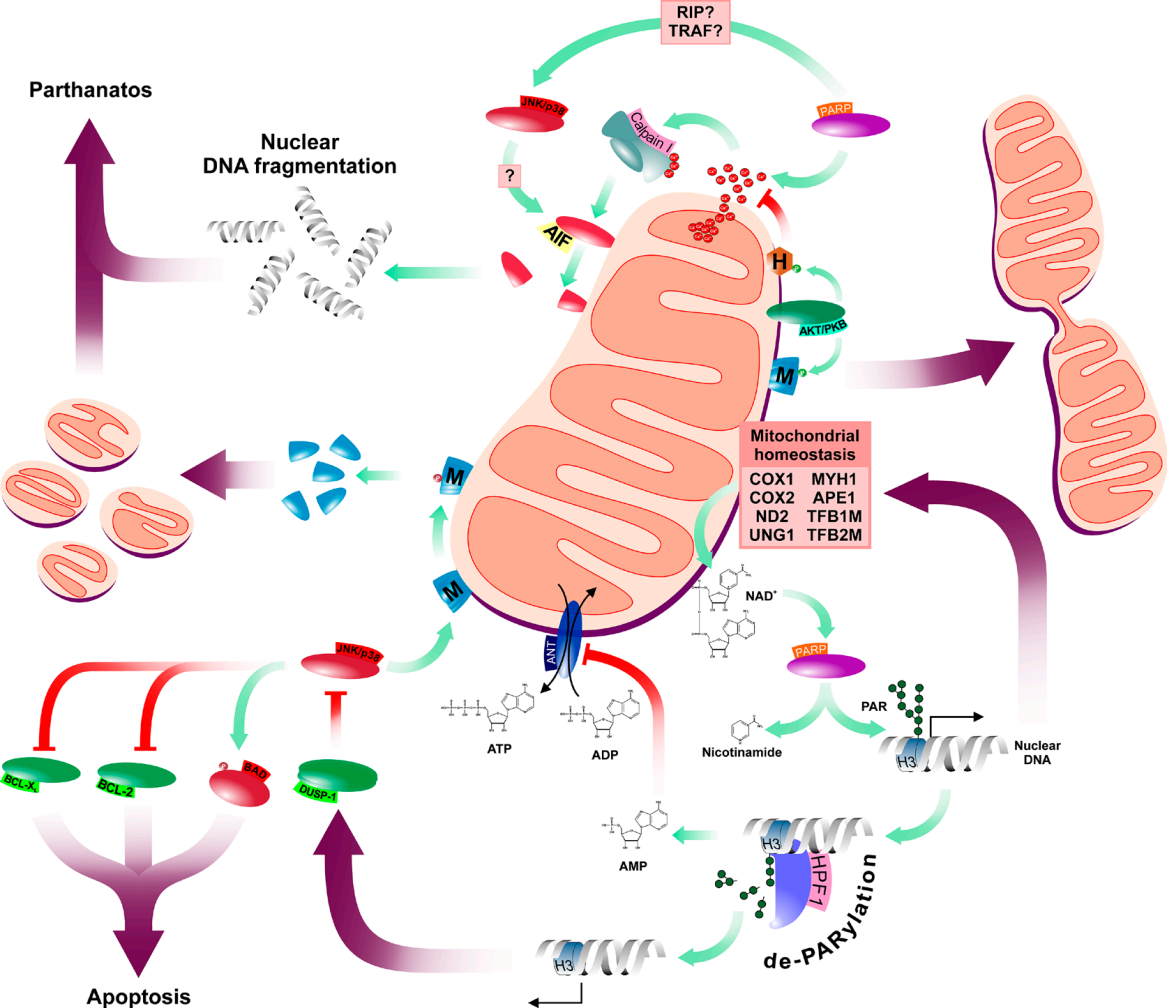
Schematic summary of PARP-1's mitochondrial functions PARP has multiple connections to the mitochondrial network affecting both mitochondrial function and morphology. Abbreviated elements are: HPF1: Histone PARylation factor 1; M: Mitofusins; H: Hexokinase II; ANT: mitochondrial adenine nucleotide translocator; H3: Histone H3; AMP, ADP and ATP: adenosine mono-, di- and triphosphate, respectively; AIF: Apoptosis Inducing Factor. Green and purple arrows represent catalysis and overall effects, respectively. Red connectors represent inhibitory interactions.

While the role of PARP-1 in various repair machineries is established, only 60% of PARP-1 complexes are detected on DNA breaks suggesting the existence of repair-independent PARP-1 functions [65]. Indeed, PARylation-mediated regulation of chromatin remodeling and transcription has been demonstrated in *Drosophila* representing a PARP-1-mediated epigenetic regulatory system [66]. In human models, this involves histone H3 and H4 that both bind PARP-1 physically. In addition to their co-localization, Histone H4 has also been shown to enhance PARP-1 activity [67]. DDR-independent recruitment of PARP-1 to chromatin and the post-translational modification of histones and DNA have been shown to induce various genes highlighting the complexity of PARP-1-mediated functions. Indeed, NAD-consuming PARP-1 contributes to the trans-activation of nuclear-encoded mitochondrial genes like the cytochrome c oxidase *COX1*, *COX2* and complex I subunit *ND2*, critical components of the mitochondrial electron transport chain. Moreover, pharmacological inhibition of PARP-1 leads to repression of the nuclear-encoded mitochondrial DNA repair factors *UNG1*, *MYH1* and *APE1* as well as mitochondrial transcription factors *TFB1M* and *TFB2M* [68]. Thus, by epigenetic marking, PARP-1 controls integrity and function of mitochondria, a critical source of PARP-1's co-enzyme NAD^+^ (Figure 3).

Besides histones, a growing number of additional molecules have also been recognized as potential targets for PARylation [69]. Systemic analysis of the PARylated proteome revealed links between PARylation and fundamental cellular processes including DNA and RNA metabolism, cell-cycle regulation, apoptosis or canonical cell signaling pathways (Supplementary Table 1). These findings suggest that the overall therapeutic effect of the pharmacological inhibition of PARP-1 may be dependent on the cellular context and further insight of PARP-1-mediated functions, in particular those affecting cellular viability, is critical for the development of safe and efficient therapeutic modalities.

### PARP-1 as a regulator of cell death

Increased PARP-1 activity leads to elevated consumption of cellular NAD^+^ that may accompanied by increased ATP consumption and, consequently, compromised energy balance, a known factor that may facilitate cell death [70, 71]. Depleted intracellular ATP pools are direct results of increased AMP levels generated by the hydrolysis of poly(ADP-ribose) which is accompanied by inhibition of the mitochondrial adenine nucleotide translocator (ANT) and reduction of mitochondrial ADP uptake and ATP release (Figure 3) [72]. Consequently, the adenylate kinase bypass, which recharges ATP from ADP, is also limited due to depleted ADP resources upon PARP-1 up-regulation [73]. Accordingly, it was hypothesized that, upon persistent PARP-1 activation, decreased mitochondrial ATP production inhibits NAD^+^ re-synthesis forming a feed-forward loop in ATP-consuming processes that, eventually, results in metabolic catastrophy and cell death. The relevance of the relationship between intracellular metabolism and PARP-1 activaton is endorsed by reports that PARP-1 activation down-regulates Hexokinase II contributing to metabolic imbalance and consequent cellular demise [74, 75].

Alternatively, PARP-1 may also reduce cellular viability via *parthanatos*. The term parthanatos originates from the abbreviation of poly(ADP-ribose) and Thanatos, the name of the God of Death in Greek mythology, and is used for a process accompanied by the loss of cell membrane integrity, large scale DNA fragmentation, phosphatidylserine externalization, dissipation of mitochondrial membrane potential, chromatin condensation and shrinkage, characteristics of a cell dying in consequence of excessive PARP-1 activation. One of the key mediators of parthanatos is the Apoptosis Inducing Factor (AIF), a caspase-independent mitochondrial death effector protein that has three putative PAR binding domains. In normal mitochondria, it shows oxido-reductase activity and functions as an anti-apoptotic factor [76]. Although the majority of AIF is anchored to the inner mitochondrial membrane, an estimated 30% is associated with the cytosolic side of the outer mitochondrial membrane [77, 78]. Upon PARP-1 activation, PAR polymers activate calpain I through mitochondrial Ca^2+^ dysregulation which leads to truncation of AIF, a critical step of its mitochondrial release and nuclear translocation. In the nucleus, PARylated AIF facilitates caspase-independent parthanatos via chromatin condensation and DNA fragmentation (Figure 3) [78–82]. Insights of the potential role of PARylation in processes like parthanatos, however, placed PARP-1, which was previously considered as a nuclear element of the DNA repair systems, into a wider cellular context and fueled further investigations on PARP-1's effects in the context of complex extranuclear systems like mitochondria.

### PARP-1 as a regulator of mitochondrial functions

PARP-1 hyperactivation is characterized by excessive nuclear NAD^+^ consumption and accompanied cytosolic NAD^+^ depletion compromising NAD^+^-dependent metabolic pathways including the glycolysis and TCA cycle that may culminate in respiratory chain malfunctions and accumulation of reactive oxygen species (ROS) [83]. It has also been documented that ROS-induced mitochondrial permeability transition may lead to intra-mitochondrial loss of NAD^+^ [84]. Thus, hyperactivation of PARP may initiates events that not only aggravates mitochondrial damage but also contribute to the ROS-mediated generation of additional single-strand DNA breaks [85]. These findings inspired the idea that PARP inhibitors might exert their cytoprotective effects by limiting the PARP-1-induced depletion of cellular NAD^+^ and, consequently, preserving mitochondrial bioenergetics. This hypothesis is underpinned by the beneficial effects of these compounds in various ischemia-reperfusion injury models, where the protective effects of PARP inhibitors are not mediated by direct antioxidant properties of the compounds used [86]. In support of this concept, PARP inhibitors were found to be effective in multiple pathophysiologic conditions including myocardial or neuronal ischemia, inflammation, oxidative stress-related cellular injury, *Diabetes mellitus* or traumatic brain injury [87–92].

Follow-up studies on PARP inhibitors using isolated mitochondria, however, suggested the existence of additional mitochondrial-resident targets with capacity to regulate mitochondrial homeostasis [93]. A potential candidate emerged from studies investigating benefits of the use of PARP inhibitor PJ-34 in septic mouse models. Using lipopolysaccharide (LPS) on PJ-34 pre-treated animals, activating phosphorylation of AKT/PKB was detected, raising the possibility that the protective effects of PARP inhibition are mediated by the AKT/PKB pathway [94]. Although the relationship between metabolism and AKT/PKB pathway has been known for decades, the mitochondrial-resident fraction of AKT/PKB was first described in 2003 [95]. Cytosolic AKT/PKB, a critical element of a highly conserved pathway activated by various stimuli in a phosphatidylinositol 3 kinase (PI3K)-dependent manner, phosphorylates a wide range of substrates including signaling pathways elements, apoptosis regulators and transcription factors involved in the regulation of cellular metabolism [96–98]. Indeed, activation of the AKT/PKB pathway leads to the induction of a number of genes involved in the glucose metabolsm including glucose transporters or the hexokinase while, directly, AKT/PKB catalyses post-translational activating phosphorylation of the phosphofructokinase [99–101]. The AKT/PKB pathway also regulates lipogenic genes by the induction of the transcription factor SREBP1c [102]. Accordingly, AKT/PKB-mediated biological responses are pleiotropic, ranging from cell survival to proliferation, intracellular trafficking or complex processes like angiogenesis [103, 104].

Similar to its cytoplasmic counterpart, the mitochondrial AKT/PKB generally exerts pro-survival effects. Indeed, translocation and activation of AKT/PKB to myocardial mitochondria enhances cardiac bioenergetics by influencing mitochondrial oxidative phosphorylation, preserves mitochondrial integrity and prevents cytochrome c release upon induction of the intrinsic apoptotic pathway [105–107]. Interestingly, however, unlike the BCL family members-mediated canonical mitochondrial pro-survival mechanisms, the anti-apoptotic function of mitochondrial AKT/PKB is glucose-dependent [108, 109]. AKT/PKB mediates its mitochondrial effects through phosphorylation of Hexokinase II, the same target that is down-regulated by PARP-1 activation. Hexokinase II phosphorylation promotes its recruitment to the mitochondrial outer membrane voltage-dependent anion channel (VDAC) [75, 108]. Upon binding to VDAC, Hexokinase II converts glucose to glucose-6-phosphate consuming mitochondrial ATP thus facilitating the carbon supply of both glycolysis and the pentose phosphate pathway [110, 111]. AKT/PKB-mediated phosphorylation of Hexokinase II also counteracts oxidant or Ca^2+^-stimulated opening of mitochondrial permeability transition pore (PTP) (Figure 3) [112].

Although detailed mechanisms are yet to be determined, observations that the angiotensin II-induced PARP-1 activation is Ca^2+^-mediated in primary culture of newborn cardiomyocytes and that PARP-1 activation follows Ca^2+^ release from perinuclear stores in depolarized primary culture of rat brain cortical neurons suggest that, at least in certain cellular contexts, Ca^2+^ may be one of the candidate mediators of PARP-1-activating stimuli [113, 114]. Thus, one might speculate that PARP inhibitors also reduce intracellular Ca^2+^ levels, possibly by utilizing or mimicking the effects of the AKT/PKB pathway thereby contributing to mitochondrial integrity and cell survival.

### PARP-1 as a regulator of mitochondrial morphology

Mitochondrial function and morphology are intimately linked. Indeed, over-expression of the mitochondrial fission proteins DRP1 or hFIS1 increases susceptibility to Ca^2+^-induced PTP opening [115]. Conversely, over-expression of the mitochondrial fusion GTP-ase Mitofusin-2 (MFN2) inhibits PTP opening [116]. Intriguingly, AKT/PKB also promotes mitochondrial fusion by activating phosphorylation of Mitofusin-1, a mitochondrial fusion-mediator transmembrane GTPase, leading to delayed onset of PTP opening and reduced cell death following ischaemia-reperfusion injury [117, 118]. OPA1, a nuclear-encoded mitochondrial dynamin-related GTPase that controls mitochondrial morphology and ultrastructure, might also be a downstream mediator of the AKT/PKB pathway [119, 120]. OPA1 is reduced under conditions associated with mitochondrial fragmentation in myocardiocytes while, upon insulin stimulation which is a known activator of the AKT/PKB pathway in the myocardium, mitochondrial fusion and OPA1 levels appear to be increased [121, 122]. The concept that AKT/PKB controls mitochondrial morphology via OPA1 is also supported by the observations that expression of both MFN2 and PARL, a protease involved in the anti-apoptotic function of OPA1, is decreased in obese and insulin-resistant patients [123, 124]. Moreover, this effect does not seem to be restricted to the myocardium since coronary endothelial cells also show reduced OPA1 levels and elevated mitochondrial fragmentation in diabetic murine models [125].

The complex effects of the AKT/PKB pathway on mitochondria is further illustrated by recent findings that AKT/PKB induces trafficking of energetically active mitochondria to the cortical cytoskeleton of tumor cells leading to lamellipodia formation, supports turnover of fatty acid complexes and random cell migration [126]. Intriguingly, this phenomenon is similar to the accumulation of mitochondria at synapses, active growth cones and branches in neuronal cells [127]. In both cases, mitochondria might provide a “regional” ATP source to fuel energy-demanding processes [126]. In support of this concept, inhibition of cellular respiration by mitochondrial DNA depletion or direct blockage of the respiratory chain prevents mitochondrial trafficking to the cortical cytoskeleton, abolishes membrane dynamics of cell motility and suppresses cell invasion [126]. Interference with MFNs suppresses mitochondrial repositioning to the cortical cytoskeleton and tumor cell invasion mediated by PI3K inhibitor therapy indicating the interplay between the AKT/PKB and MFN pathways [126]. Hence, one can speculate that alterations in mitochondrial morphology and function promoted by the AKT/PKB pathway represent another mechanism of the observed mitoprotective effects of PARP inhibitors.

The AKT/PKB pathway, however, is not the only signaling mechanism associated with the regulation of mitochondrial morphology and PARP-1. Activation of JNK and p38 MAP kinases has also been reported in response to PARP-1 and both kinases are involved in the regulation of mitochondrial dynamics and function (Figure 3) [128–130]. The potential mediator of this effect is the dual specific protein phosphatase-1 (DUSP-1). Indeed, PARP-1 inhibition attenuates JNK and p38 through the increased expression of DUSP-1 enhancing cells survival [131]. JNK phosphorylates BCL-2 and BCL-X_L_, attenuating their pro-survival activity that facilitates cytochrome c release and collapse of the mitochondrial membrane potential [132]. It can also directly trigger the intrinsic apoptotic pathway by phosphorylating the pro-apoptotic BCL-2 family member BAD [133]. Apart from the regulation of the canonical intrinsic apoptotic pathway, JNK actively contributes to the mitochondrial morphology as well. JNK-mediated phosphorylation of MFN2 promotes its proteasomal degradation by the E3 ubiquitin ligase HUWE1 leading to mitochondrial fragmentation and enhanced apoptotic cell death [134]. Conversely, blockade of the Transferrin Receptor 1 (TFR1)-JNK pathway reduces HUWE1-mediated MFN2 ubiquitination preserving the fused mitochondrial network and function [135]. Similarly, pharmacological inhibition of p38 biases mitochondrial dynamics toward fusion and maintains mitochondrial functions [136].

Despite the apparent contribution of JNK and p38 to the regulatation of mitochondrial morphology and functions, both proximal and distal elements of the putative PARP-1-JNK/p38 axis remain to be identified. As for proximal mediators, RIP and TRAF were suggested to convey signals between PARP-1 and JNK while the observation that PARP-1-induced JNK activation is indispensable for mitochondrial depolarization, AIF translocation and subsequent cell death suggests that AIF might act as an effector of the PARP-1-TRAF/RIP-JNK/p38 pathway [128, 137].

This model predicts cytoprotective effects of PARP inhibitors in tissues like skeletal muscle or neural ones that highly dependent on mitochondrial functions. In support of this concept, PARP inhibition is apparently beneficial in a number of muscle dysfunction models [138, 139]. Moreover, PARP inhibition attenuated the mitochondrial toxin cuprizone-induced oligodendrocyte depletion and demyelination in experimental models. These mitoprotective effects are believed to be mediated by suppression of JNK and p38 phosphorylation, increased activation of the AKT/PKB pathway and repression of apoptosis [140].

### PARP-1 as a regulatior of inflammation

**Figure 4:**
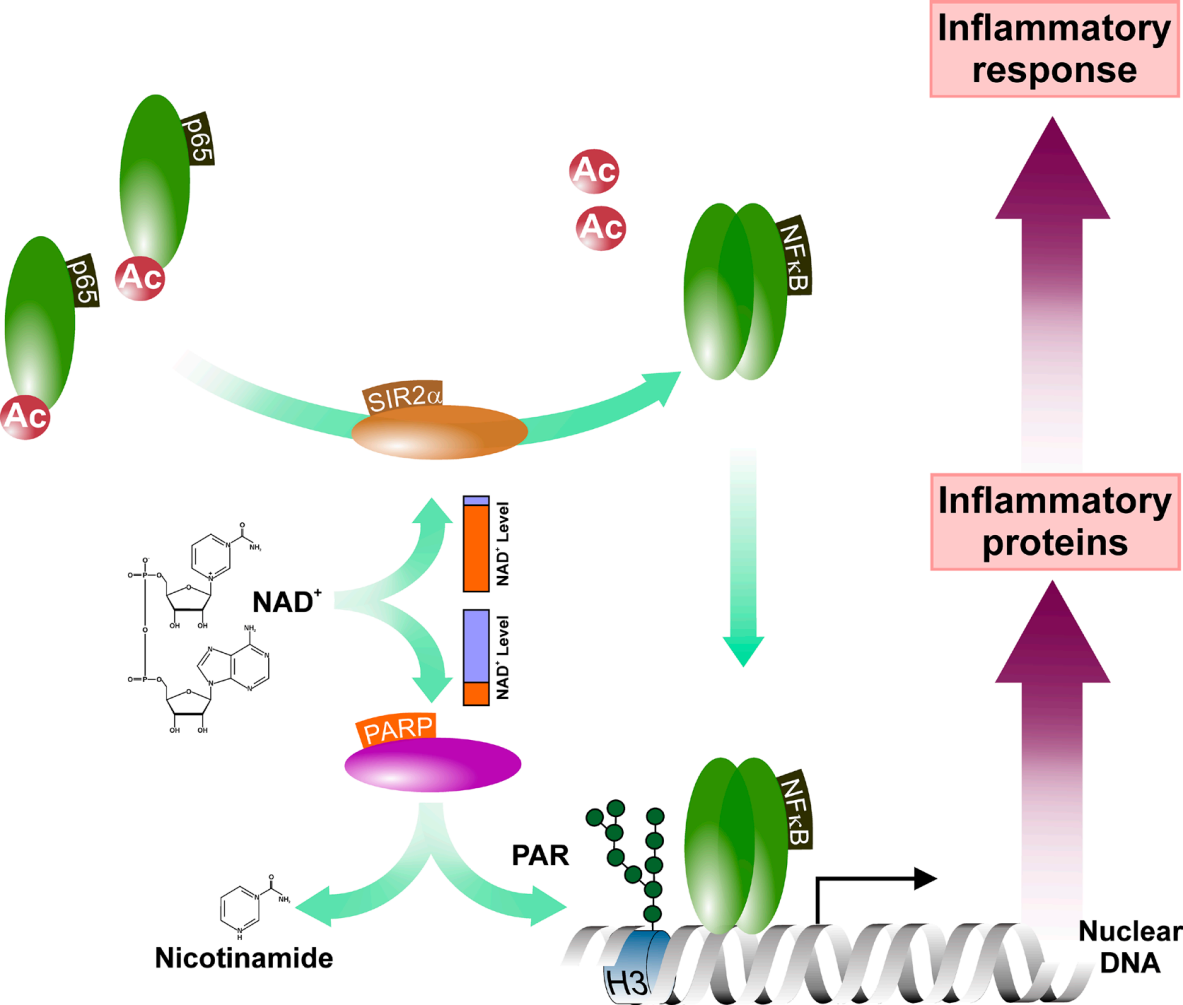
PARP activity influences the NF-κB-mediated inflammatory signaling The PARP activation-mediated depletion of the NAD^+^ pools attenuates cellular NAD-dependent systems including the sirtuins. This may affect the rate of deacetylation of, among others, the NF-κB subunit p65 and, thus, influence the NF-κB-mediated inflammatory gene expression. Abbreviated elements are: Ac: Acetyl-group; H3: Histone H3; PAR: poly(ADP-ribose) polymer; SIR2α: NAD-dependent protein deacetylase Sirtuin-1.

Protective effects of PARP inhibitors in disease models of acute lung inflammation or septic shock predict a role for PARP-1 in inflammation as well [29, 141]. Indeed, analysis of PARP-1^−/−^ mice revealed increased resistance to LPS-induced endotoxic shock and failed induction of NF-κB-regulated inflammatory genes due to the missing co-activator function of PARP-1 [142–144]. Interestingly however, although both lung and liver equally responsive to LPS-mediated NF-κB activation, PARP-1 inhibition disrupts the NF-κB-mediated response to LPS in the liver only suggesting the existence of tissue-specific elements in the PARP-1/NF-κB interactome [94]. One of the candidate interactors is Sir2alpha (SIR2α) that directly targets NF-κB (Figure 4). SIR2α is a primarily nuclear-resident, NAD^+^-dependent protein deacetylase with a wide range of intracellular targets including signaling molecules like AKT/PKB, protein components of the chromatin or transcription factors and their co-regulators. Since the K_m_ of PARP-1 for NAD^+^ falls in the low micromolar range, PARP-1 may influence SIR2α activity by reducing NAD^+^ bioavailability [145–147]. Indeed, depletion of cellular NAD^+^ levels upon PARP-1 activation reduces SIR2α deacetylase activity. Conversely, reduced PARP-1 activity increases intracellular NAD^+^ levels and enhances SIR2α activity leading to SIR2α-mediated deacetylation and induction of mitochondrial biogenesis [148, 149]. Intriguingly, PARP-2 also interacts with SIR2α directly down-regulating the *SIR2α* promoter [150].

Based on the Human Integrated Protein Expression Database, the SIR2α polypeptide is most enriched in lung tissues. It has also been reported that acetylation reduces the DNA-binding affinity of RELA/p65 [151]. Thus, one can speculate that elevated SIR2α activity may activate NF-κB upon LPS treatment in PARP-inhibited lung tissues while, conversely, the low relative abundance of SIR2α in hepatocytes leads to disruption of the inflammatory responses in the liver. This hypothesis also suggests that PARP inhibition-based anti-inflammatory modalities may not be efficient in SIR2α over-expressing tissues like prostate, fetal heart, testis or lymphocytes but may be potent in inflammed organs in which the putative SIR2α-mediated bypass mechanism is not present. In support of this concept, PARP inhibition was found to reduce TNFα induced inflammatory responses of synovial fibroblasts suggesting the potential use of PARP inhibitors in conditions like rheumatoid arthritis [152]. The idea that SIR2α may rescue NF-κB activity upon PARP inhibitions also raises the question whether PARP-1 functions as a transcriptional co-factor of the inflammatory pathways by regulating deacetylation of NF-κB via recruitment of deacetylases or preventing the interaction between acetylases and NF-κB.

Although these questions need further investigations, detailed analysis of the PARP-1/NF-κB interactome holds out the promise to discover novel, potentially tissue-specific targets of future anti-inflammatory therapies. Indeed, pharmacological inhibition of PARP reverted elevated colonic permeability and reduced water absorption in a chronic colitis model using IL-10-deficient mice [153]. Down-regulation of pro-inflammatory gene expression observed in a PARP-1-depleted mouse enterocolitis model upon exposure to *Salmonella typhimurium* suggests that these effects might be related to the NF-κB mediated gene regulatory functions of PARP-1 [154]. Reduction of the NF-κB activation and pro-inflammatory gene expression observed in a PARP-1^−/−^ negative mouse model of contact dermatitis further supports this concept [155]. Similarly, repression of pro-inflammatory cytokines upon PARP-1 inactivation by either genetic ablation or pharmacologic inhibition might also explain compromised recruitment of inflammatory cells observed in a pulmonary inflammation model. These data may also open the door to the use of PARP inhibitors in pathophysiologic conditions like *Diabetes mellitus*. Indeed, PARP inhibitors efficiently reduce hyperglycemia-induced NF-κB activation and *in vivo* podocyte depletion, a hallmark of diabetic glomerulopathy [156].

### Conclusions and perspectives

On the ground of discovery of PARP-1 and its role in the DNA repair machinery, PARP inhibition emerged as a novel therapuetic concept to eradicate cancer cells. This concept was further fueled by the discovery of the mitoprotective nature of PARP inhibitors, raising the possibility of reduced side effects on the non-transformed surrounding tissue [86, 157]. What we have learned from both *in vitro* and *in vivo* studies on PARP inhibition, however, revealed a more complex picture and prompts reconsideration of the possible and effective use of PARP inhibitors in the clinical practice. Indeed, the periodic increase of PAR activity during cell cycle highligths the importance of PARylation not only in the S-phase but also in the M-G1 transition and might serve as one of the critical factors in the regulation of migration and, potentially, formation of metastases [158, 159]. In addition, the typical mitochondrial effects of PARP inhibition including membrane potential maintenance, reduced oxygen/glucose consumption and lower intracellular concentrations of ROS and ATP are similar to that of cancer cells raising concerns about the systemic use of PARP inhibitors [160].

Data showing activation of the AKT/PKB pathway upon PARP inhibition may also be alarming. By inactivation of glycogen synthase kinase-3, caspase-9, BAD or the forkhead homologue rhabdomyosarcoma transcription factor, AKT/PKB has been shown to be a critical factor of cytostatic resistance of transformed cells [161]. Indeed, AKT/PKB promotes chemo-resistance of cells by regulating the ABC transporter BCRP1 activity which enhances drug efflux. In addition, in prostate cancer cells, AKT/PKB was shown to mediate effects of the metastasis-associated gene 1 that promotes cellular transformation and metastasis generation via regulation of E-cadherin [162–165]. Similar findings have been reported in terms of JNK and SIR2α showing their role in drug resistance and induction of metastases, respectively [166, 167]. Thus, one may be concerned that PARP inhibitors may also exert similar effects by activating the AKT/PKB, JNK or SIR2α pathways. Indeed, PARP inhibition-induced taxol resistance has already been reported to be independent of the intracellular NAD^+^ level but mediated by the AKT/PKB pathway [161]. Although these findings may be worrying, they also suggest that simultaneous pharmacologic inhibition of PARP-1 and AKT/PKB may be a more efficient and, more importantly, a safer strategy for targeting PARP-1 in future antineoplastic chemotherapies. This concept is supported by the recent report that the use of pharmacologic inhibitors of the repair kinase ATM apparently bypasses chemoresistance for PARP-inhibitors in BRCA-ablated cells [168].

Insight into the extranuclear effects of PARP-1 raised the possibility of the use of PARP inhibitors in a wider range of human pathologies as well. Inhibition of PARP-1 leads to tyrosine phosphorylation of the vascular endothelial growth factor receptor-2 (VEGFR2), activation of AKT/PKB and disruption of NF-κB-mediated inflammation adding new aspects to the therapeutic use of these compounds in chronic inflammatory conditions like vascular disease or *Diabetes mellitus* [143, 169].

The unexpectedly tangled cellular effects of PARP clearly call for further investigations. These efforts not only hold out the promise of enhancement of the therapeutic concept of PARP inhibition but a better understanding of the complex PARP-1 interactome as well.

## SUPPLEMENTARY MATERIALS TABLES





## Abbreviations

ABC transporter: ATP-binding cassette transporters
ABC transporter BCRP1: ATP-binding cassette sub-family G member 2 Breast cancer resistance protein
AIF: Apoptosis Inducing Factor
APE1: DNA-(apurinic or apyrimidinic site) lyase 1
BAD: BCL-2-associated agonist of cell death
BCL-2: B Cell Lymphoma-2
BER: Base-excision repair
BRCA1/2: Breast cancer type 1/2 susceptibility protein
BRCT: Breast Cancer-Associated 1 C-terminal domain
COX1: Cytochrome C oxidase 1
COX2: Cytochrome C oxidase 2
DBM: DNA-binding module of PARP-1
DDB2: DNA damage-binding protein 2
DDR: DNA damage response
DRP1: Dynamin-1-like protein
DUSP-1: Dual specific protein phosphatase-1
EXO1: MutSα-exonuclease 1
hFIS1: Human mitochondrial fission 1 protein
HPF1: Histone PARylation factor 1
HUWE1: Huwe1 E3 ubiquitin ligase
JNK: c-Jun N-terminal kinase 1
LPS: Lipopolysaccharide
LPS: Lipopolysaccharides
mANT: Mitochondrial adenine nucleotide translocator
MAP kinase: Mitogen-activated protein kinase
MFN2: Mitofusin-2
mPTP: Mitochondrial permeability transition pore
MYH1: Adenine DNA glycosylase
NAD^+^: Nicotinamide adenine dinucleotide
NER: Nucleotide excision repair
NF-κB: Nuclear factor NF-kappa-B
NHEJ: Non-homologous end joining repair
NLS: Nuclear localisation signal
OPA1: Optic Atrophy Protein 1
PARG: Poly(ADP-ribose) glycohydrolase
PARP: Poly(ADP-ribose) polymerases
PARP: Poly-adenosine diphosphate-ribose polymerase
PCNA: Proliferating cell nuclear antigen
PI3K: Phosphatidylinositol 3-kinase
PSARL: Presenilins-associated rhomboid-like protein
RELA/p65: V-Rel Reticuloendotheliosis Viral Oncogene Homolog, Nuclear factor NF-kappa-B p65 subunit
RIP: Receptor-interacting serine/threonine-protein kinase 1
RNF146: E3 ubiquitin-protein ligase RNF146, Iduna
ROS: Reactive oxygen species
SIR2α: Sir2alpha
SREBP1c: Sterol regulatory element binding protein 1c
TFB1M: Dimethyladenosine transferase 1, mitochondrial
TFB2M: Dimethyladenosine transferase 2, mitochondrial
TFR1: Transferrin Receptor 1
TNFα: Tumor necrosis factor alpha
TRAF: Tumor necrosis factor type 2 receptor-associated protein 3
UNG1: Uracil-DNA glycosylase
VDAC: Voltage-dependent anion channel
VEGFR2: Vascular endothelial growth factor receptor-2
XRCC1: X-ray repair cross complementing protein 1.
