# Supplementary material for: Poly(adenosine diphosphate-ribose) polymerase as therapeutic target: lessons learned from its inhibitors
Source: Oncotarget. 2017 Apr 5;8(30):50221–39. doi: 10.18632/oncotarget.16859 (PMC5564845; doi:10.18632/oncotarget.16859)
Supplement: Supplementary file 2 [file oncotarget-08-50221-s002.docx]

**Supplementary Table 1: Summary of the identified members of the PARP-1 interactome.**See Supplementary_Table_1
